# Proteome turnover in bacteria: current status for *Corynebacterium glutamicum* and related bacteria

**DOI:** 10.1111/1751-7915.12035

**Published:** 2013-02-20

**Authors:** Christian Trötschel, Stefan P Albaum, Ansgar Poetsch

**Affiliations:** 1 Department of Plant Biochemistry, Ruhr-University Bochum44780, Bochum, Germany; 2 Computational Genomics, Center for Biotechnology (CeBiTec), Bielefeld University33615, Bielefeld, Germany; 3 Biodata Mining Group, Faculty of Technology, Bielefeld University33615, Bielefeld, Germany

## Abstract

With the advent of high-resolution mass spectrometry together with sophisticated data analysis and interpretation algorithms, determination of protein synthesis and degradation rates (i.e. protein turnover) on a proteome-wide scale by employing stable isotope-labelled amino acids has become feasible. These dynamic data provide a deeper understanding of protein homeostasis and stress response mechanisms in microorganisms than well-established ‘steady state’ proteomics approaches. In this article, we summarize the technological challenges and solutions both on the biochemistry/mass spectrometry and bioinformatics level for turnover proteomics with a focus on chromatographic techniques. Although the number of available case studies for *Corynebacterium glutamicum* and related actinobacteria is still very limited, our review illustrates the potential of protein turnover studies for an improved understanding of questions in the area of biotechnology and biomedicine. Here, new insights from investigations of growth phase transition and different stress dynamics including iron, acid and heat stress for pathogenic but also for industrial actinobacteria are presented. Finally, we will comment on the advantages of integrated software solutions for biologists and briefly discuss the remaining technical challenges and upcoming possibilities for protein turnover analysis.

## General aspects of protein turnover in bacteria

Standard transcriptomics and proteomics experiments detect the mRNA or protein level, but this value is the result of two, often simultaneously occurring processes, namely synthesis and degradation. Ignoring this fact greatly hampers the understanding of the intrinsically dynamic processes governing life, which affects questions from basic (e.g. regulatory networks) to applied research (e.g. strain optimization). One of the first technologies for tracing newly synthesized proteins and calculating protein turnover was radioactive labelling, which still remains the common pulse-chase experiment. Even before the advent of powerful proteome separation techniques like 2D-electrophoresis and the invention of biological mass spectrometry, the simple pulse-chase analysis of total cellular protein delivered a fundamental insight into bacterial physiology: a generally low protein degradation rate for growing bacteria experiencing ideal nutrient supply (Larrabee *et al*., 1980), and substantial reuse of amino acids through proteolysis under hunger conditions (Pine, 1972), which may reach up to 30% h^−1^ of total intracellular protein. These observations were confirmed in newer, more sophisticated proteome studies for the bacterial pathogen *Staphylococcus aureus* (Michalik *et al*., 2009). Here, almost no protein synthesis was found in the stationary phase, and especially anabolic enzymes were quite rapidly degraded with the onset of the stationary phase. In our own studies with *Corynebacterium glutamicum*, we detected overall very low protein degradation rates under ambient temperature (30°C) and even under heat stress (40°C) (Trotschel *et al*., 2012) during exponential growth. From this admittedly still small number of examples, the impression emerges that under ideal environmental conditions bacteria do not require protein degradation in large quantities to sustain optimal growth. Such knowledge is also relevant for industrial processes, since product formation is often started in the early stationary phase. Another point worth mentioning with regard to standard proteomics and transcriptomics experiments, is the often reported weak correlation between abundance changes on the mRNA and protein level. Under the assumption of a high protein synthesis rate and almost no protein degradation during exponential growth phase, one could expect better correlation between transcriptome and proteome, whereas in the stationary phase significant differences should emerge. Indeed, in our time-course analysis of the adaptation to hyperosmotic stress (Franzel *et al*., 2010), transcriptome and proteome showed for the majority of gene products the same trends. Of note is a shift to later time-points for significant changes in the proteome, which may be due to the more dynamic nature of the transcriptome and the preceding transcription before protein synthesis. These findings are corroborated by recent protein turnover studies for *Bacillus subtilis* focusing on the stationary phase that reported a drastic increase in proteolysis during glucose starvation (Gerth *et al*., 2008).

In the following sections, various aspects of proteome turnover studies will be presented and; technological challenges and solutions, case studies in actinomycetes, our recently developed protein turnover technology platform for *C. glutamicum*, and anticipated future trends in this field.

## Technological challenges and solutions

### Experimental approaches

#### 2D-electrophoresis for turnover determination

One of the first technologies for tracing newly synthesized proteins and calculating protein turnover was radioactive labelling. In the common pulse-chase experiments (Takahashi and Ono, 2003), the biological material (e.g. bacteria) is cultivated in the absence of a (radioactive) tracer, and at a defined time-point the tracer (e.g. a radioactive amino acid) is added and consumed (pulse phase); thereupon, the sample is cultivated again without the tracer and its disappearance, for instance due to protein degradation, is monitored (chase phase). The technology has already been applied from microorganisms to mammals since about 40 years (Pine, 1972; Larrabee *et al*., 1980). Advantageous are the extremely high sensitivity of radiation detection and the rather low investment costs compared with alternative technologies. However, use of radioactivity requires containment measures and hampers or even precludes additional experimental procedures, for instance protein identification with mass spectrometry. Since only cumulative values for overall cellular protein synthesis and degradation rates are far from satisfactory, protein separation methods that are compatible with radioactive labelling have been sought for. The most successful combination has been radioactive pulse-labelling with 2D-electrophoresis, where each sample is separated usually on two identical gels: one gel for staining with mass spectrometry (MS)-compatible reagents (e.g. Coomassie) for protein identification, the other gel for protein quantification, where the radioactive signal is related to an added radioactive standard. One application example is the study of bacterial proteome adaptation during exposure to antibiotics for discovery of their mode of action (Bandow and Hecker, 2007). Another application is combined radioactive labelling with ^14^C and ^35^S to monitor the physiological adaptation of bacteria during transition into the stationary phase (Michalik *et al*., 2009). The latter work is also one of the earliest examples, where both protein synthesis and degradation rates could be determined. In order to circumvent the use of radioactive material, protein pulse-labelling with SILAC (Doherty *et al*., 2009) (^13^C lysine, ^13^C arginine, ^15^N) was combined with 2D-electrophoresis – here a uniform internal standard for calculation of protein synthesis and degradation was obtained by ^15^N-labelling of all amino acids (Michalik *et al*., 2012). Although 2D-electrophoresis is compatible with radioactive labelling or SILAC and suitable for turnover determinations, owing to its well-known limitations (membrane proteins, extreme pI, low protein abundance …) (Gygi *et al*., 2000), gel-free technologies for the study of proteome turnover have been developed.

#### Chromatography-based solutions

Proteome separation without 2D-gels involves almost always chromatographic separation of a proteolytic digest, usually using reverse-phase chromatography (RPLC) due to its superior resolution and compatibility with electrospray ionization (ESI) MS. However, complex proteomes require at least two separation dimensions, for instance a combination of strong cation exchange and reverse-phase chromatography, the ‘MudPIT’ technology (Wolters *et al*., 2001), or SDS-PAGE and chromatographic separation of proteolytic digests from gel slices (Stone *et al*., 1998). Since for several elements heavy stable isotopes exist (e.g. ^2^H, ^13^C, ^15^N), it has become feasible to pulse with these stable isotopes and use MS both for protein identification and quantification to determine *de novo* synthesis, proteolysis, or protein turnover, i.e. the synthesis over degradation ratio (reviewed in Doherty and Beynon, 2006). Although labelling is mainly carried out by feeding heavy amino acids (e.g. ^2^H_10_ Leu, ^13^C_6_ Lys) (Pratt *et al*., 2002), feeding microorganisms with cheaper compounds such as ^13^C_6_ glucose or ^15^NH_4_Cl is often a good alternative (Cargile *et al*., 2004). The foundation for turnover quantification with this approach is the ability of MS to easily discriminate light from heavy molecules. While various bacterial examples for protein turnover profiles with stable isotope labelling have been published, a common theme is the utilization of precursor metabolite combinations (e.g. ^12^C_6_Lys/^13^C_6_Lys) from a very small set of molecules: For turnover analysis of *Mycobacterium smegmatis* under acid shock and iron starvation conditions growth on ^15^N-medium (Rao *et al*., 2008b) was used for labelling. The same group utilized ^15^N-arginine to monitor protein turnover in *Mycobacterium tuberculosis*. Another trick to obtain protein synthesis and degradation data was to use a combination of SILAC and iTRAQ-labelling [iTRAQ is a chemical label that allows relative quantification on the MS/MS-level (Aggarwal *et al*., 2006)]. In this experimental design, SILAC was used as pulse-label and iTRAQ for comparison of different samples (time-points) in a single MS run, which allowed to determine protein degradation rates (Jayapal *et al*., 2010) for *Streptomyces coelicolor*. Apart from its high price, the iTRAQ approach suffers from a limited dynamic range and peak overlaps on the MS and MS/MS level, which can lead to false quantification values. (Rao and colleagues, 2008a) published another strategy aiming at the parallel determination of synthesis and degradation based on MS chromatogram alignment. They obtained a dataset for *Mycobacterium smegmatis* covering several time-points after a heavy isotope pulse. Such an alignment of MS chromatograms is a common approach for label-free proteomics (reviewed in Wang *et al*., 2008), but for various reasons (peak overlaps, matrix effects …) it is still less accurate than label-based approaches. Even though RPLC-ESI does not possess many of the aforementioned limitations of 2D-electrophoresis, it comes with higher demands for MS hardware and data analysis. Recently, an alternative label-based approach was described. It was observed that compared with radioactive labelling turnover determination with stable isotopes is very difficult for early time-points. This is due to the fact that in the early stage isotope label incorporation is low and, as a consequence, signals from newly synthesized proteins may be below the instrument detection level. Signals may, also be obscured by overlapping molecule species, which may even be the unlabelled form of the same peptide. In order to circumvent these problems, labelling with unnatural amino acids has been developed (Dieterich *et al*., 2006). For example, the different chromatographic behaviour of methionine vs the methionine analogue azidohomoalanine was exploited (Kramer *et al*., 2009). Whereas turnover rates of some hundred proteins could be determined for early time-points in *Escherichia coli*, extension to incubation times above 30 min was impossible due to physiological impairment caused by azidohomoalanine, and as another drawback only methionine containing peptides could be quantified.

### Software solutions for turnover calculation

The degree of computational challenges for analysis of mass spectrometry data depends on the labelling strategy – the higher the expected spectral complexity and overlap, the more demanding will be the task of protein identification and quantification. Unfortunately, global labelling with simple nitrogen or carbon compounds (e.g. ^13^C-glucose), albeit counter intuitive, puts the highest demands on the data analysis. This is due to the fact that the more atoms could be potentially labelled, the more isotopologs may be observed in the MS spectrum. Therefore, the earliest automatic analyses of protein turnover were done with SILAC labelling and software that was originally developed for label-based relative quantification. One early example is the SILAC-based quantification feature of the software MaxQuant (Cox and Mann, 2008), which has been used for turnover analysis (Selbach *et al*., 2008). For SILAC quantification, the software locates light/heavy pairs in an MS chromatogram based on expected mass differences. Afterwards, these pairs are scored according to the expected isotope intensity distribution and the observed peak intensity. As the labelling is limited to specific SILAC amino acids, typically lysine and/or arginine, data analysis is greatly simplified. However, the application of this approach is restricted to organisms that are auxotroph for the labelled amino acid(s), and furthermore, only peptides containing at least one labelled amino acid can be quantified at all. For 15N metabolic labelling, older programs assuming fixed isotope incorporation ratios, like RelEx (MacCoss *et al*., 2003) and ProRata (Pan *et al*., 2006), have also been used in my group for proteome turnover calculations. Reasonable turnover ratios can be calculated with these programs as long as the isotope incorporation level does not vary a lot between all heavy and light peptides in one sample. As soon as substantial variance can be expected, which should always be assumed for global amino acid labelling, this must be accounted for in data analysis. First efforts trying to consider this fact employed manual or semi-automatic calculation of isotope distributions for different incorporation levels (Snijders *et al*., 2005; Rao *et al*., 2008a; Sperling *et al*., 2008). Owing to this time-consuming procedure, turnover was calculated for a smaller number of proteins only. A first software tool that calculates and considers fractional isotope labelling was QuantiSpec (Haegler *et al*., 2009). The possibility to process peptides with varying isotope incorporation values without restrictions to amino acid composition makes it more universally applicable than MaxQuant, with the price of a more complicated spectral analysis and less reliable quantification. This condition is quite well approximated for later time-points post pulsing, but early after pulsing the isotope incorporation levels display a wide distribution. To work around the problem of low label incorporation levels, the developers of QuantiSpec put forth a different approach that does not require determination of labelling level in the software ProTurnyzer (Zhang *et al*., 2011). They demonstrated for *E. coli* that in larger peptides ≥ 15 amino acids, the monoisotopic peak almost disappears for ≥ 20% total ^15^N incorporation and thus allows calculating the amount of unlabelled peptide in mixed labelled/unlabelled isotope patterns. Lastly, it should be mentioned that the group of Burlingame recently presented a data analysis pipeline for ^15^N-labelling (Guan *et al*., 2011), which according to data for mice, should be powerful, but has not found application for microorganisms yet.

## Case studies

Protein dynamics is still a much neglected aspect in the field of proteomics research. Predominantly, quantitative mass spectrometry is being employed to capture a snapshot of the cellular proteome at any given time-point – in that a relative and/or absolute quantification are carried out to determine alterations in abundance or absolute protein copy-number. There are, currently, several publications existing that deal with protein turnover in four different species belonging to the order *Actinomycetales*: (i) transition from exponential growth to stationary phase in antibiotics producer *Streptomyces coelicolor*, (ii) iron starvation and acid shock in model strain *Mycobacterium smegmatis*, (iii) iron starvation in the human pathogen *Mycobacterium tuberculosis* and (iv) salt stress, growth on different carbon sources as well as heat adaptation in the amino acid producer *C. glutamicum* (Jayapal *et al*., 2008; 2010; Rao *et al*., 2008a,b; Franzel *et al*., 2010; Trotschel *et al*., 2012; Voges and Noack, 2012). All these species belong to the same class, *Actinobacteria*, a very large and diverse group of Gram-positive, high G + C bacteria (Ventura *et al*., 2012). Depending on 16S rDNA/rRNA sequence-based phylogenetic clustering, strains of this class can consistently be recovered as members of the same phylogenetic lineage, revealing > 80% sequence similarity among each other (Stackebrandt *et al*., 1997). As part of the suborder *Corynebacterineae C. glutamicum*, *M. smegmatis* and *M. tuberculosis* are closely related, whereas *S. coelicolor* belongs to the suborder *Streptomycineae*. These bacteria are important for the production of bio-(active) compounds, cause of human diseases, or in case of *C. glutamicum*, *M. smegmatis* and *S. coelicolor* serve as model organisms for *M. tuberculosis*.

### (i) Growth phase transition in *S. coelicolor*

Streptomycetes are among the most numerous and ubiquitous soil bacteria and exhibit a complex growth cycle consisting of filamentous vegetative growth, formation of aerial hyphae and differentiation of these into spore chains. Notably, strains of this group are utilized for the production of pharmaceutically relevant compounds including anti-tumour agents, immunosupressants and over two-thirds of currently used natural antibiotics. With 8.7 Mb and 7825 predicted genes, *S. coelicolor* is the bacterium with the largest sequenced genome (Bentley *et al*., 2002). Notably, this bacterium has a remarkably high G + C content of 72.1%. The group of Wei-Shou Hu (Jayapal *et al*., 2008; 2010) investigated protein dynamics during the stationary phase adaptation in *S. coelicolor*. This process is of immense interest as it pertains to physiological transformations inducing biosynthesis of the above mentioned naturally occurring therapeutic agents. Another work underscores the high relevance of proteome turnover studies to understand differentiation of this microorganism, since time series experiment with relative iTRAQ quantification have pointed out the occurrence of differentiation processes in liquid cultures used for industrial fermentations that are comparable to solid, sporulating cultures (Manteca *et al*., 2010).

In their first publication, Jayapal and colleagues (2009) investigated the proteome of *S. coelicolor* using iTRAQ-labelling and performed a transcriptome comparison, in an effort to gain insight into the transition from exponential to stationary growth. This obviously implies, one needs to sample at multiple time-points to warrant ample resolution over time, safeguarding full dynamics of this specific process. In total, eight samples at t = 7, 11, 14, 16, 22, 26, 34 and 38 h were taken and, subsequently, labelled with iTRAQ. As the iTRAQ system can only compare four samples in one experiment, all samples were combined in such a way that, in the end, three measurements allowed comparing all eight samples. This strategy yielded 1100 identified proteins, representing proteome coverage of 14%. Of these proteins, 330 were identified in all sampled time-points and could, hence, supply full protein dynamics over time. Protein classification showed primarily nucleotide biosynthesis-related proteins (80% of all proteins identified), ribosomal subunits (76%) and to a lesser extend amino acid biosynthesis-related (43%), central metabolism intermediate (40%) and energy metabolism (39%). Proteins of other subclasses were only scarcely represented. A so-called codon adaptation index (CAI) was established for all genes with respect to ribosomal genes, to identify possible increases in protein expression that correlate with optimal codon usage. Indeed, proteins with high CAI could be identified more often. For a proteome to transcriptome comparison, all data were scaled to time-point 1, i.e. 7 h, to allow for a comparison of relative changes and to investigate possible correlations. For all samples, in total, a Pearson's correlation coefficient (*r*) of 0.63 was determined; limiting this to the highest 50 and lowest 50 genes, however, with respect to their CAI-value, an *r*-value of 0.8 and 0.35 was calculated respectively. Hence, genes having a low CAI-value displayed comparatively low correlation. With the help of principal component analysis (PCA), this trend in mRNA and protein dynamics was investigated.

In this analysis, in total, 798 gene-products were covered for which at least four time-points were available. Missing time-points were estimated using linear interpolation. Astonishingly, prominent patterns in both protein and mRNA domains correlated rather well, including energy metabolism (glycolysis, TCA-cycle and pentose phosphate cycle), secondary metabolism as well as actinorhodin-synthesis, nucleic acid and protein synthesis, a stress response with catalases and alkyl hydroperoxide reductases. In contrast, 36% of all mRNA-protein pairs displayed different time-courses in the PCA, with 1/3 giving rise to opposite trends. Identification of differences between mRNA and protein dynamics might have important biological implications as they indicate discrepancies in synthesis rates and stability of proteins and mRNA as well as possible post-transcriptional regulation. For the glutamate-uptake system GluABCD but also for the xylose-uptake system a clear increase in protein abundance was observed during the stationary phase, whereas the corresponding transcripts were downregulated. A further interesting group consisted of the ATPase subunits and RNA-polymerases, displaying essentially stable protein levels while corresponding mRNA amounts declined rapidly during the stationary phase. Taken together, genes with an optimal codon usage displayed a better correlation between mRNA and protein abundance, indicating the notion that genes are regulated at the transcription level. Regulation at the post-transcriptional level appears to take place for genes with a lower codon usage.

Jayapal's initial study aimed at understanding the time-resolved dynamics of protein synthesis and breakdown in *S. coelicolor*. In an effort to segregate these two underlying mechanisms, a novel multitagging approach was developed based on stable isotope tagging (SILAC) and isobaric tag for relative and absolute quantification (iTRAQ) labelling (Jayapal *et al*., 2010). To this end, *S. coelicolor* was first incubated in minimal medium containing [^13^C_6_,^15^N_4_] labelled arginine. Following at least 5–6 doublings, cells were harvested and taken up in minimal medium lacking labelled arginine and cultivated into the stationary phase. Four samples were taken at t = 0, 2, 4 and 8 h and peptides obtained during these time-points were additionally labelled with iTRAQ to capture dynamic shifts in growth rates. Samples of all four time-points were pooled and analysed using mass spectrometry. In MS1 the light isotope could be detected apart from its heavy counterpart. Every signal contained the respective peptides following the SILAC-pulse at all four time-points. Upon CID fragmentation, the iTRAQ-tag signal intensity was measured in MS2 to determine the decay in labelled proteins. Using this approach, protein degradation rates for a total of 115 proteins could be estimated. Observed rates were, for example, at 0.056 and 0.049 h^−1^ for phosphoglycerate kinase and enolase giving rise to the most stable signals. Two other glycolysis proteins, glyceraldehyde 3-phosphate dehydrogenase and glucose 6-phosphate isomerase, with breakdown rates of 0.083 and 0.102 h^−1^ respectively, were considered moderately stable. Furthermore, two pyruvate kinase isoforms displayed opposite degradation rates with Pyk2 being rather stable at 0.059 h^−1^, whereas Pyk1 rated at 0.171 h^−1^ displaying much higher degradation. Pyk2 is considered to function as primary housekeeping pyruvate kinase, which may possibly explain its low degradation rates. Subunits of the ATPase were also identified and γ-, α- and β-subunit degradation was 0.063, 0.090 and 0.115 h^−1^, respectively, again displaying considerable variation within one single enzyme complex. Two methionine biosynthesis enzymes also displayed differential degradation rates, in that the putative methionine synthase (MetE) had rather high degradation rates of 0.130 h^−1^, whereas adenosylhomocysteinase displayed much lower rates (0.052 h^−1^). Protein stability is influenced by a multitude of external factors, such as protein molecular weight, surface area, pI as well as amino acids composition. However, Jayapal and co-workers were unable to identify significant correlations between protein degradation and the above outlined factors in *S. coelicolor*. This methodology allows for the selective detection of newly synthesized and ‘old’ proteins due to the multitagging procedure. It is hence possible to quantify protein degradation in non-steady state systems, accomplished by iTRAQ labelling.

Classical proteomics studies applied to *S. coelicolor* indiciate processes that may be relevant for adjusting protein turnover. For instance, Ser/Thr/Tyr protein phosphorylation has been reported to occur most extensively during transition into sporulation (Manteca *et al*., 2011) and the identified different phosphorylation of transcription factors may among other proteins affect protein synthesis rates.

### (ii) Iron starvation and acid shock in *M. smegamatis*

*Mycobacterium smegmatis* is a saprophytic, fast-growing soil dweller, also to be found in water and plants. Due to being akin to the human pathogen *M. tuberculosis* and its ease of genetic modification, this species is currently being used as model organism (Shiloh and DiGiuseppe Champion, 2010). The 6.99 Mb genome of *M. smegmatis* contains 6938 genes with a relatively high GC-content of 67% (submitted 19 October 2006 to the Institute for Genomic Research, Rockville, MD). Rao and colleagues (2008b) analysed protein turnover in cells that were (i) placed in slightly acidic medium (pH = 5) as compared with standard medium (pH = 7), and (ii) grown in the presence of higher iron concentrations. Upon infecting the human body, *M. tuberculosis* is exposed to multiple stressors during phagocytosis, a process initialized by macrophages, such as reactive oxygen species (ROS), lytic enzymes, and nutrient deficiency (Voskuil *et al*., 2003). The acid-stress experiment used minimal medium in which *M. smegmatis* was incubated up to mid-log phase to subsequently add ^15^N-ammonium sulfate. The neutral pH culture served as control. Following one doubling, cells were harvested. The iron-deficiency experiment consisted of two replicates being cultivated, with or without FeCl_3_, in the presence of the ^15^N isotope, as soon as the mid-log phase was reached; the medium was exchanged for a minimal medium containing the ^14^N-isotope. Subsequently, Rao *et al*. detected 247 proteins in four samples; 114 proteins were present in both stress cultures; 121 proteins were present only in the iron-stress cultures. In total, 84 proteins in all four samples could be identified. There were 151 proteins with synthesis-over-degradation ratio (S/D) determined in at least one pair of cell cultures in the same experiment (pH stress or iron-starvation). Their approach is capable of measuring S/D over a dynamic range of three orders of magnitude, in which the ATP synthase β chain in the acid shocked culture showed the lowest value at 0.18 and glycerol kinase in the high-iron control culture the highest value at 521. Interestingly, protein turnover correlation profiles between stressed and control samples gave a Pearson's correlation coefficient of 0.27, whereas the iron-starvation experiment gave a higher coefficient of 0.81. This suggests that the S/D for many proteins in the pH 5 cells underwent significant readjustment in different directions, while the S/D for the most proteins remained similar or shifted in the same direction when the cells were shifted from high- to low-iron media. Of the 84 proteins detected in all four cultures, 31 had statistically significant S/D changes in both the acid shock and iron-starvation experiment and had S/D changed by more than twofold in at least one of the two comparisons. Interestingly, turnover increased under acid shock for 28 of the 31 proteins but decreased under iron-starvation for all the 31 proteins. Many of these targets may have coordinated response to stresses due to their role in common processes affected by the stresses. For example, 12 proteins are involved in transcription and translation, three further are related to oxidative stress response. Of the 31 proteins the catalase/peroxidase KatG, possibly involved in neutralizing the reactive oxidant peroxynitrous acid, and the ATPase F1 β subunit AtpD were the only two proteins with a significant decrease in turnover in both conditions. Consequently, the oxidative stress response and ATP synthesis may be negatively influenced. Remarkably, the foremost antituberculosis agent isoniazid requires activation by KatG to be bactericidal (Timmins *et al*., 2004b) leading to the generation of oxidative species (Timmins *et al*., 2004a). This ultimately results in mycobacterial dormancy, isoniazide, however, kills off only growing mycobacteria and is ineffective against those being dormant.

### (iii) Iron starvation in *M. tuberculosis*

Opposed to all other actinomycetes described so far, *M. tuberculosis* is a human pathogenic bacterium which mainly invades lung tissue. In 2010, there were 8.8 million incident cases of tuberculosis, of which approximately 1.5 million died (http://www.who.int/tb/publications/global_report/2011/gtbr11_full.pdf). The 4.41 Mb genome contains 3995 genes with a GC-content of 66% (Camus *et al*., 2002). As in the aforementioned experiment on *M. smegmatis* Rao *et al*. analysed iron starvation dependent protein turnover in this species (Rao *et al*., 2008a). Therefore, iron-starved late-log-phase *M. tuberculosis* cells were diluted in fresh low- and high-iron media containing [^15^N]-asparagine for protein labelling upon dilution. After 24 h of cultivation the two samples were harvested and used for both relative quantification illustrating changes in protein abundance and protein turnover yielding synthesis over degradation ratios (S/D). By the extracted ion chromatographic (XIC) intensity the abundance of each isotopomer in an isotopologue was estimated. Every peptide is represented by two isotopologues: one originating from the [^14^N]-labelled pre-cultured fraction, and a further one being *de novo*-synthesized after the [^15^N] pulse. The intensities of all [^14^N]- as well as [^15^N]-isotopologues of a protein were summed up and together with the sum of both values the relative changes in protein abundance between high-iron and low-iron cells were calculated. The protein turnover S/D was determined using the ratio of the [^15^N]- to the [^14^N]-isotopologues. Finally, 104 proteins for protein abundance as well as turnover in both high-iron and low-iron *M. tuberculosis* cells were quantified. Though, proteins could be included that were in a dynamic range of four orders of magnitude in XIC intensity. The stress protein GroEL2 with a value of 0.29 had the highest XIC intensity suggesting that this target has a high abundance under the chosen conditions. In contrast, ModD, a protein with unknown function, exhibits the lowest intensity with 4.5 × 10^−5^. Of the 104 proteins, only five proteins were upregulated in the high-iron medium compared with the low-iron medium, while 16 proteins were downregulated. For example, the three proteins PpiA, KatG and Rv0284 showed on both the proteome and transcriptome level (Rodriguez *et al*., 2002) the same characteristics. While the expression of PpiA and KatG was increased, Rv0284 was significantly less abundant. The analysis of the protein synthesis over degradation ratios (S/D) illustrated, that there was a more active protein synthesis in the high-iron medium. In the low-iron cells 24 proteins showed a significant increase in S/D, while eight proteins had a significant decrease. In contrast, in the high-iron cells 56 proteins significantly increased in S/D, whereas only five proteins showed an S/D decrease. Subsequently, in a cluster analysis 101 of the 104 proteins could be attached to three different groups summarizing their behaviour. Group 1 includes the proteins that in general showed an increase in XIC intensities as well as S/D. For example eight ribosomal proteins are listed in this group, what generally suggests an increased protein synthesis in the high-iron medium. In group 2, there were those proteins that showed overall a decrease in the XIC intensities, but had various changes in S/D. Group 3 comprises proteins with an increase in XIC intensities for the *de novo*-synthesized targets, but a decrease of the intensities of the [^14^N] isotopologues resulting in a strong increase of the S/D-values. The two stress related proteins superoxide dismutase (SodA) and catalase-peroxidase-peroxynitritase T (KatG), both are key members of the mycobacterial reactive oxidative species detoxification system, are part of this group. In the high-iron medium the synthesis of these two enzymes using iron as a cofactor increased significantly. These results are consistent with the fact that a high-iron load in the host increases virulence of *M. tuberculosis in vivo*, because the oxidative stress response is improved.

### (iv) Growth on an alternative carbon source and salt as well as heat stress response in *C. glutamicum*

Like Streptomycetes, *C. glutamicum* is a soil bacterium that is used for biotechnological production. Kinoshita and colleagues (2004) isolated *C. glutamicum* during a screening programme for new glutamate producing bacteria in Japan. Annually, industrial scale bioreactors produce 2.0 million tons of glutamate additive and 1.5 million tons of the feed additive lysine (Becker and Wittmann, 2012). With 3.28 Mb and 3058 genes *C. glutamicum* possesses a rather small genome (Kalinowski *et al*., 2003) and the G + C content is lower (53.8%) as compared with other Actinomycetes.

#### Salt stress

In its natural habitat, but also during fermentation processes, *C. glutamicum* is exposed to various stresses, with osmotic stress being considered rather important. For over two decades the hyperosmotic salt-stress has been investigated in *C. glutamicum* at a molecular level. Transporter and biosynthesis pathways have been identified and characterized that supply compatible solutes (Kramer and Morbach, 2004; Weinand *et al*., 2007). Such solutes, as for example proline and betaine, counteract water loss upon stress induction and this was studied at a more global level (Franzel *et al*., 2010) using quantitative proteomics and transcriptomics. *Corynebacterium glutamicum* was stressed with sodium chloride during the exponential phase of growth. This resulted in a short lag-phase followed by a period of reduced growth rate (during which further samples were taken for analysis). For relative quantification purposes an external ^15^N-standard was added and predominantly membrane and membrane attached proteins were analysed using SIMPLE [specific integral membrane peptide level enrichment (Fischer *et al*., 2006)]. Analysis of cytosolic, membrane-associated, and membrane proteins using MudPIT [multidimensional protein identification technology, (Washburn *et al*., 2001)] technology resulted in 1058 identified proteins (35% proteome coverage) of which 822 proteins could be quantified. Compared with the stress-free control culture, 111 proteins displayed significant regulation and the stress-induced uptake systems ProP for prolin and ectoin were strongly upregulated (20-fold) at the protein level and transcriptome level (fourfold). Furthermore, the uptake system BetP for betaine and glycine displayed 2.7- and 3.0-fold upregulation respectively. Osmocarriers LcoP and EctP were, surprisingly, not regulated and the majority of proteins involved in lipid and cell wall biosynthesis, were also upregulated in response to the stress. These enzymes are expected to alter bacterial membrane composition, as underpinned by lipidome studies in the same study. At the level of the transcriptome, acetyl/propionyl-CoA carboxylase β chain DtsR1, butyryl-CoA: acetate coenzyme A transferase ActA and biotin carboxyl carrier protein AccBC were all significantly downregulated. Downregulation was, furthermore, observed for sulfur metabolism-related proteins and genes, whereas homocysteine methyltransferase MetE, O-acetylserine (thiol)-lyase CysK, cystathionine γ-synthase MetB, O-acetylhomoserine-thiol lyase MetY and ferredoxin-sulfite reductase CysI were all upregulated at the protein level, but downregulated at the transcriptome level, emphasizing the importance of post-transcriptional regulation in these systems.

#### Growth on acetate and glucose

*Corynebacterium glutamicum* can grow on organic acids such as acetate and lactate, also in the presence of glucose. Growth on acetate is relevant with respect to growth under oxygen-limited conditions, giving rise to increased acetate levels – that are subsequently taken up and metabolized. Central carbon metabolism during glucose and acetate usage was analysed in-depth by Voges and Noack (2012) using a selected reaction monitoring (SRM) mass spectrometry-based approach, allowing for both relative as well as absolute quantification. The required isotope-labelled internal standard was produced by cultivating *C. glutamicum* on minimal medium containing not the ^14^N-isotope but rather the heavy ^15^N-isotope. In total, 19 enzymes of the central carbon metabolism were selected and time-resolved alterations were monitored during (i) growth on glucose or acetate and (ii) an acetate pulse upon glucose starvation. Dynamic adaptation of enzymes to alterations in carbon source or growth-phase was detected using targeted SRM mass spectrometry with three respective reporter peptides per protein being quantified using an externally spiked labelled internal peptide standard. With glucose or acetate as sole carbon source, samples were taken during (i) mid-exponential growth, (ii) the exponential to stationary phase transition and (iii) after 2 h cultivation during the stationary phase. Relative protein changes were expressed with respect to the glucose growth condition. This yielded no significant changes in enzyme concentration over time during the various growth-phases, excluding malate dehydrogenase and menaquinone oxidoreductase, which displayed up- and downregulation respectively. In stark contrast, however, changes in carbon source from glucose to acetate significantly lowered the expression of glycolysis enzymes, irrespective of the growth-phase under study. During cultivation on acetate, both glyoxylate shunt/bypass enzymes isocitrate lyase and malate synthase were more abundant during cultivation on acetate – an observation that is also supported by transcriptomics studies (Muffler *et al*., 2002). Moreover, the abundance of aconitase was increased, supplying the isocitate lyase substrate. To charter dynamic modulation of *C. glutamicum*'s proteome, cultivation on glucose was performed initially, followed by a starvation phase and an acetate pulse. Interestingly, this regime halted cellular growth and aconitase, isocitrate lyase, malate synthase and phosphoenolpyruvate carboxykinase increased significantly during the initial 90 min of incubation but decreased upon 2 h of acetate starvation. Taken together, highly abundant proteins are apparently used to safeguard cell survival during growth on alternative carbon sources.

### A new platform for protein turnover studies and its application to the *C. glutamicum* heat stress response

**Figure 1:**
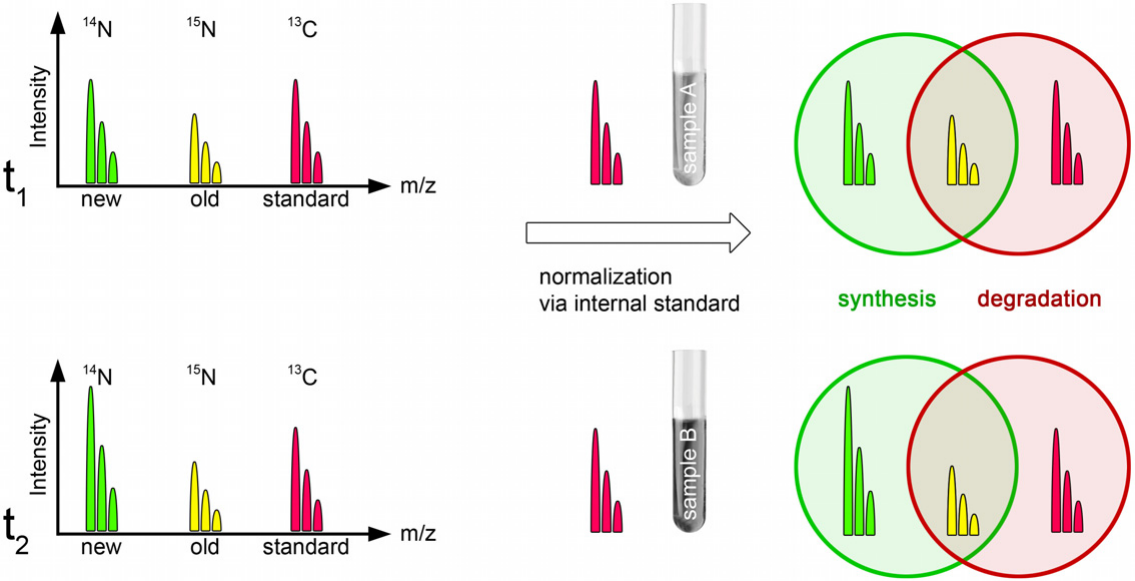
Multilabelling approach using both ^15^N and ^13^C for isotopic labelling. The *C. glutamicum* cells were cultivated on ^15^N enriched medium and then transferred to minimal medium containing only the ^14^N source. After label switch the ‘new’ proteins – in contrast to the previously synthesized ‘old’ proteins – incorporate the light isotope. In addition, a ^13^C standard was generated and spiked to the pulsed samples. At the end, for every peptide three different isotopes exist. After normalization the ratio between the ^14^N and ^15^N peptides indicates the degree of synthesis whereas the ratio between the ^15^N and the ^13^C isotope results in the degree of degradation.

**Figure 2:**
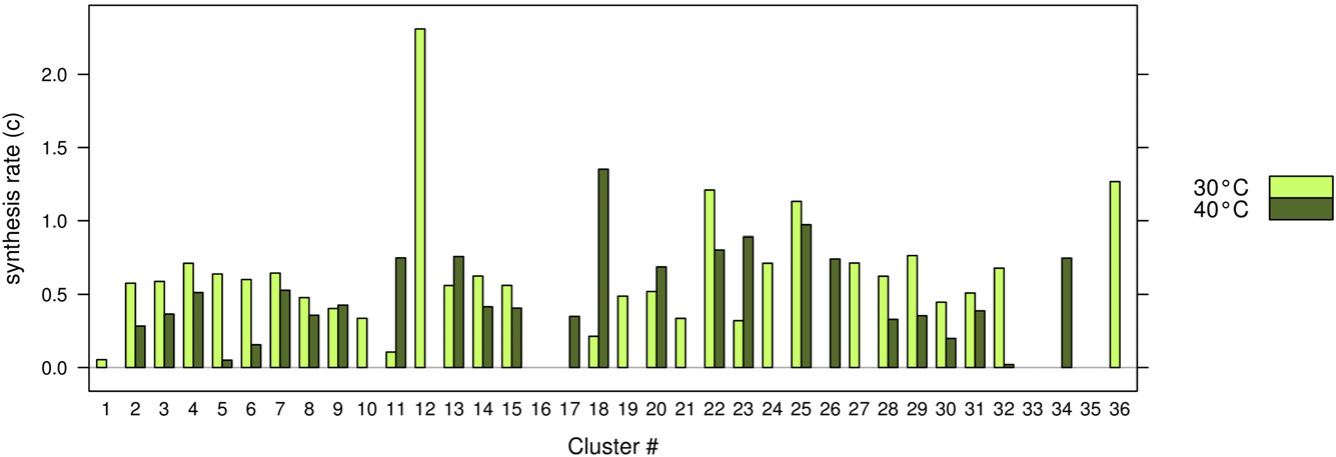
Average protein synthesis for all clustered proteins. The light green bars represent the synthesis rate at 30°C, the dark green bars stand for the synthesis rate at 40°C. The synthesis rate (c) is derived by non-linear regression [y = a − b e^(−c x)^]. Missing bars indicate that the number of values was too low for a rate calculation.

**Figure 3:**
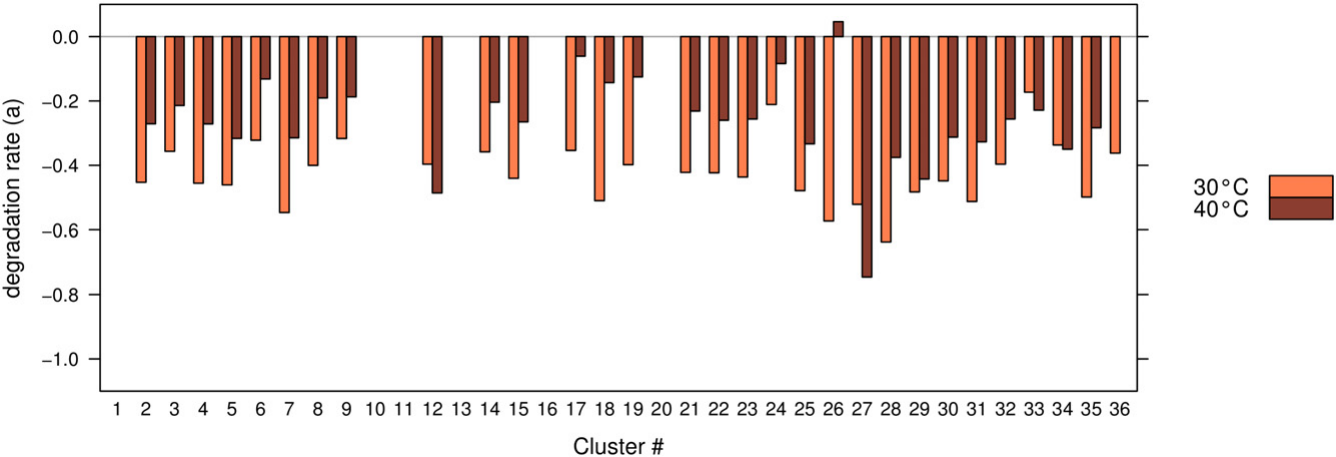
Average protein degradation for all clustered proteins. The light red bars represent the degradation rate at 30°C, the dark red bars stand for the degradation rate at 40°C. The degradation rate (a) is derived by linear regression [y = a x + b]. Missing bars indicate that the number of values was too low for a rate calculation.

Unsatisfied by existing platforms for bacterial protein turnover analysis and the lack of biological data, we put efforts into the development of a flexible multilabelling method that gives access to protein-turnover, *de novo* synthesis and degradation on the proteome scale for *C. glutamicum* (Trotschel *et al*., 2012). For the first time, it was now possible to examine protein synthesis and degradation separately in actinomycetes. To this end, *C. glutamicum* was cultivated in minimal medium with ^15^NH_4_Cl as sole nitrogen source. Incorporation of the heavy isotope was maintained until the maximal rate of protein synthesis was reached. At an enrichment rate of ≥ 98%, a ^14^N-Pulse was applied by a culture medium exchange. At this time-point newly synthesized proteins incorporated the light N-isotope, allowing for specific detection of ‘old’ (heavy) and ‘new’ (light) proteins. *De novo* protein synthesis could now be estimated by comparing the respective peak-ratios (see Fig. 1 for a description of the procedure). Concurrently, ^13^C-glucose was added to incorporate heavy carbon isotopes. The obtained protein sample was subsequently used as an internal standard to be added to pulse chase cultivations, allowing for the calculation of protein degradation using the ^15^N-peak as reference (Fig. 1). This ^15^N enriched synthesized protein could only be altered by means of degradation. The internet application QuPE (Albaum *et al*., 2009) was developed to calculate protein synthesis and breakdown, and also has the ability to securely store, statistically analyse and visualize obtained data, e.g. using cluster-analysis. Special care was taken with respect to the incorporation of heavy-N into newly synthesized protein. The initial ^15^N-pool will eventually be replaced but was initially incorporated into newly synthesized proteins as well. Verification was performed by combining labelled with unlabelled samples with defined ^14^N/^15^N ratios, allowing for exact calculation of incorporation rates. For a proof of principle, heat-stress was selected as well-characterized and experimentally controllable bacterial adaptation process. *Corynebacterium glutamicum* was cultivated at 30°C in the presence of the ^15^N-isotope and upon the transition into the exponential phase heat-stress was applied to one culture while simultaneously setting a ^14^N pulse to both. Next, samples were taken at 30, 60, 120 and 210 min and analysed with and without ^13^C internal standard. Apart from cytosolic protein, membrane proteins were analysed in-depth. In total, 805 proteins were identified (∼ 25% genome coverage), with 85% of these passing quantification criteria (698 proteins). Taken together, *de novo* synthesis is higher at 30°C as compared with 40°C, albeit that both cultivations had similar growth-rates. Hierarchical cluster analysis was used to identify co-regulated proteins. In total 36 clusters could be determined grouping, for example, several heat-shock-related proteins, ribosomal proteins, and ATPase subunits each into one cluster. The resulting cluster information is then used to calculate cluster-wide synthesis and degradation rates using linear or non-linear, respectively, regression (Fig. 2 and Fig. 3). Heat-shock protein synthesis was, as expected, upregulated at 40°C, with the majority of *de novo* synthesis taking place during the first 30 min following heat-stress application. For other proteins, synthesis at 40°C was significantly reduced, as for example for ATPase subunits (cluster 32 in Fig. 2). With respect to protein degradation at 30 and 40°C, rates for 158 and 156 proteins could be estimated respectively (Fig. 3). Subsequently, 79 and 93 proteins were degraded at 30 and 40°C, with thiol-disulfide isomerase and methionine synthase II showing enhanced degradation. Heat-shock, hence, resulted in significantly higher protein degradation rates, as shown previously (Araki, 1992) for other species, too.

### Current challenges and possible future trends for protein turnover studies

By comparison of the different approaches for analysing protein turnover, it becomes obvious that none is universally applicable. Each approach has its own strengths and weaknesses, and one must carefully design the experiment according to the scientific question. If the growth rate (i.e. isotope label incorporation) is significantly slower than the studied biological process, techniques performing better with low label incorporation are most suitable, while, on the opposite, approaches allowing monitoring on a longer time range should be favoured. Ideally, in future, a software solution will come up that flexibly adjusts the isotope pattern analysis strategy to the automatically determined isotope incorporation level. Concerning protein synthesis, combined transcriptome and protein turnover analysis has the potential to reveal novel regulation mechanisms; yet such studies (Maier *et al*., 2011) are very rare and often time-consuming due to insufficient data (analysis) integration. Concerning proteolysis, the influence of post-translational modifications on protein degradation has been neglected in proteome turnover investigations. There are ongoing works in several groups towards lifting relative quantification to absolute quantification for proteome turnover and we should expect such reports in the next couple of years. Given the availability and maturity of proteome turnover technology that more groups will be motivated to conduct such dynamic proteome studies either as new investigations or re-investigations of scientific questions for which static proteome data has already been obtained. Having said this, we hope that our recently introduced platform solution will inspire many future protein turnover studies for *C. glutamicum* and related bacteria.

## References

[b18] Aggarwal K, Choe LH, Lee KH (2006). Shotgun proteomics using the iTRAQ isobaric tags. Brief Funct Genomic Proteomic.

[b54] Albaum SP, Neuweger H, Franzel B, Lange S, Mertens D, Trotschel C (2009). Qupe – a rich internet application to take a step forward in the analysis of mass spectrometry-based quantitative proteomics experiments. Bioinformatics.

[b55] Araki T (1992). An analysis of the effect of changes in growth temperature on proteolysis in vivo in the psychrophilic bacterium Vibrio sp. strain ANT-300. J Gen Microbiol.

[b8] Bandow JE, Hecker M (2007). Proteomic profiling of cellular stresses in *Bacillus subtilis* reveals cellular networks and assists in elucidating antibiotic mechanisms of action. Prog Drug Res.

[b47] Becker J, Wittmann C (2012). Bio-based production of chemicals, materials and fuels – *Corynebacterium glutamicum* as versatile cell factory. Curr Opin Biotechnol.

[b37] Bentley SD, Chater KF, Cerdeno-Tarraga AM, Challis GL, Thomson NR, James KD (2002). Complete genome sequence of the model actinomycete *Streptomyces coelicolor* A3(2). Nature.

[b44] Camus JC, Pryor MJ, Medigue C, Cole ST (2002). Re-annotation of the genome sequence of *Mycobacterium tuberculosis* H37Rv. Microbiology.

[b16] Cargile BJ, Bundy JL, Grunden AM, Stephenson JL (2004). Synthesis/degradation ratio mass spectrometry for measuring relative dynamic protein turnover. Anal Chem.

[b24] Cox J, Mann M (2008). MaxQuant enables high peptide identification rates, individualized p.p.b.-range mass accuracies and proteome-wide protein quantification. Nat Biotechnol.

[b22] Dieterich DC, Link AJ, Graumann J, Tirrell DA, Schuman EM (2006). Selective identification of newly synthesized proteins in mammalian cells using bioorthogonal noncanonical amino acid tagging (BONCAT). Proc Natl Acad Sci USA.

[b14] Doherty MK, Beynon RJ (2006). Protein turnover on the scale of the proteome. Expert Rev Proteomics.

[b9] Doherty MK, Hammond DE, Clague MJ, Gaskell SJ, Beynon RJ (2009). Turnover of the human proteome: determination of protein intracellular stability by dynamic SILAC. J Proteome Res.

[b51] Fischer F, Wolters D, Rogner M, Poetsch A (2006). Toward the complete membrane proteome: high coverage of integral membrane proteins through transmembrane peptide detection. Mol Cell Proteomics.

[b5] Franzel B, Trotschel C, Ruckert C, Kalinowski J, Poetsch A, Wolters DA (2010). Adaptation of *Corynebacterium glutamicum* to salt-stress conditions. Proteomics.

[b6] Gerth U, Kock H, Kusters I, Michalik S, Switzer RL, Hecker M (2008). Clp-dependent proteolysis down-regulates central metabolic pathways in glucose-starved *Bacillus subtilis*. J Bacteriol.

[b32] Guan S, Price JC, Prusiner SB, Ghaemmaghami S, Burlingame AL (2011). A data processing pipeline for mammalian proteome dynamics studies using stable isotope metabolic labeling. Mol Cell Proteomics.

[b11] Gygi SP, Corthals GL, Zhang Y, Rochon Y, Aebersold R (2000). Evaluation of two-dimensional gel electrophoresis-based proteome analysis technology. Proc Natl Acad Sci USA.

[b30] Haegler K, Mueller NS, Maccarrone G, Hunyadi-Gulyas E, Webhofer C, Filiou MD (2009). QuantiSpec – quantitative mass spectrometry data analysis of (15)N-metabolically labeled proteins. J Proteomics.

[b33] Jayapal KP, Philp RJ, Kok YJ, Yap MG, Sherman DH, Griffin TJ, Hu WS (2008). Uncovering genes with divergent mRNA-protein dynamics in *Streptomyces coelicolor*. PLoS ONE.

[b19] Jayapal KP, Sui S, Philp RJ, Kok YJ, Yap MG, Griffin TJ, Hu WS (2010). Multitagging proteomic strategy to estimate protein turnover rates in dynamic systems. J Proteome Res.

[b48] Kalinowski J, Bathe B, Bartels D, Bischoff N, Bott M, Burkovski A (2003). The complete *Corynebacterium glutamicum* ATCC 13032 genome sequence and its impact on the production of L-aspartate-derived amino acids and vitamins. J Biotechnol.

[b46] Kinoshita S, Udaka S, Shimono M (2004). Studies on the amino acid fermentation. Part 1. Production of L-glutamic acid by various microorganisms. J Gen Appl Microbiol.

[b23] Kramer G, Sprenger RR, Back J, Dekker HL, Nessen MA, van Maarseveen JH (2009). Identification and quantitation of newly synthesized proteins in *Escherichia coli* by enrichment of azidohomoalanine-labeled peptides with diagonal chromatography. Mol Cell Proteomics.

[b49] Kramer R, Morbach S (2004). BetP of *Corynebacterium glutamicum*, a transporter with three different functions: betaine transport, osmosensing, and osmoregulation. Biochim Biophys Acta.

[b1] Larrabee KL, Phillips JO, Williams GJ, Larrabee AR (1980). The relative rates of protein synthesis and degradation in a growing culture of *Escherichia coli*. J Biol Chem.

[b26] MacCoss MJ, Wu CC, Liu H, Sadygov R, Yates JR (2003). A correlation algorithm for the automated quantitative analysis of shotgun proteomics data. Anal Chem.

[b56] Maier T, Schmidt A, Guell M, Kuhner S, Gavin AC, Aebersold R, Serrano L (2011). Quantification of mRNA and protein and integration with protein turnover in a bacterium. Mol Syst Biol.

[b38] Manteca A, Jung HR, Schwammle V, Jensen ON, Sanchez J (2010). Quantitative proteome analysis of *Streptomyces coelicolor* nonsporulating liquid cultures demonstrates a complex differentiation process comparable to that occurring in sporulating solid cultures. J Proteome Res.

[b39] Manteca A, Ye J, Sanchez J, Jensen ON (2011). Phosphoproteome analysis of Streptomyces development reveals extensive protein phosphorylation accompanying bacterial differentiation. J Proteome Res.

[b3] Michalik S, Liebeke M, Zuhlke D, Lalk M, Bernhardt J, Gerth U, Hecker M (2009). Proteolysis during long-term glucose starvation in *Staphylococcus aureus* COL. Proteomics.

[b10] Michalik S, Bernhardt J, Otto A, Moche M, Becher D, Meyer H (2012). Life and death of proteins: a case study of glucose-starved *Staphylococcus aureus*. Mol Cell Proteomics.

[b53] Muffler A, Bettermann S, Haushalter M, Horlein A, Neveling U, Schramm M, Sorgenfrei O (2002). Genome-wide transcription profiling of *Corynebacterium glutamicum* after heat shock and during growth on acetate and glucose. J Biotechnol.

[b27] Pan C, Kora G, McDonald WH, Tabb DL, VerBerkmoes NC, Hurst GB (2006). ProRata: a quantitative proteomics program for accurate protein abundance ratio estimation with confidence interval evaluation. Anal Chem.

[b2] Pine MJ (1972). Turnover of intracellular proteins. Annu Rev Microbiol.

[b15] Pratt JM, Petty J, Riba-Garcia I, Robertson DH, Gaskell SJ, Oliver SG, Beynon RJ (2002). Dynamics of protein turnover, a missing dimension in proteomics. Mol Cell Proteomics.

[b20] Rao PK, Rodriguez GM, Smith I, Li Q (2008a). Protein dynamics in iron-starved *Mycobacterium tuberculosis* revealed by turnover and abundance measurement using hybrid-linear ion trap-Fourier transform mass spectrometry. Anal Chem.

[b17] Rao PK, Roxas BA, Li Q (2008b). Determination of global protein turnover in stressed mycobacterium cells using hybrid-linear ion trap-Fourier transform mass spectrometry. Anal Chem.

[b45] Rodriguez GM, Voskuil MI, Gold B, Schoolnik GK, Smith I (2002). *ideR*, an essential gene in mycobacterium tuberculosis: role of IdeR in iron-dependent gene expression, iron metabolism, and oxidative stress response. Infect Immun.

[b25] Selbach M, Schwanhausser B, Thierfelder N, Fang Z, Khanin R, Rajewsky N (2008). Widespread changes in protein synthesis induced by microRNAs. Nature.

[b40] Shiloh MU, DiGiuseppe Champion PA (2010). To catch a killer. What can mycobacterial models teach us about *Mycobacterium tuberculosis* pathogenesis?. Curr Opin Microbiol.

[b28] Snijders AP, de Koning B, Wright PC (2005). Perturbation and interpretation of nitrogen isotope distribution patterns in proteomics. J Proteome Res.

[b29] Sperling E, Bunner AE, Sykes MT, Williamson JR (2008). Quantitative analysis of isotope distributions in proteomic mass spectrometry using least-squares Fourier transform convolution. Anal Chem.

[b36] Stackebrandt E, Rainey FA, Ward-Rainey NL (1997). Proposal for a new hierarchic classification system, Actinobacteria classis nov. Int J Syst Bacteriol.

[b13] Stone KL, DeAngelis R, LoPresti M, Jones J, Papov VV, Williams KR (1998). Use of liquid chromatography-electrospray ionization-tandem mass spectrometry (LC-ESI-MS/MS) for routine identification of enzymatically digested proteins separated by sodium dodecyl sulfate-polyacrylamide gel electrophoresis. Electrophoresis.

[b7] Takahashi M, Ono Y (2003). Pulse-chase analysis of protein kinase C. Methods Mol Biol.

[b42] Timmins GS, Master S, Rusnak F, Deretic V (2004a). Requirements for nitric oxide generation from isoniazid activation in vitro and inhibition of mycobacterial respiration in vivo. J Bacteriol.

[b43] Timmins GS, Master S, Rusnak F, Deretic V (2004b). Nitric oxide generated from isoniazid activation by KatG: source of nitric oxide and activity against *Mycobacterium tuberculosis*. Antimicrob Agents Chemother.

[b4] Trotschel C, Albaum SP, Wolff D, Schroder S, Goesmann A, Nattkemper TW, Poetsch A (2012). Protein turnover quantification in a multilabeling approach: from data calculation to evaluation. Mol Cell Proteomics.

[b35] Ventura M, Canchaya C, Tauch A, Chandra G, Fitzgerald GF, Chater KF, van Sinderen D (2007). Genomics of Actinobacteria: tracing the evolutionary history of an ancient phylum. Microbiol Mol Biol Rev.

[b34] Voges R, Noack S (2012). Quantification of proteome dynamics in *Corynebacterium glutamicum* by (15)N-labeling and selected reaction monitoring. J Proteomics.

[b41] Voskuil MI, Schnappinger D, Visconti KC, Harrell MI, Dolganov GM, Sherman DR, Schoolnik GK (2003). Inhibition of respiration by nitric oxide induces a *Mycobacterium tuberculosis* dormancy program. J Exp Med.

[b21] Wang M, You J, Bemis KG, Tegeler TJ, Brown DP (2008). Label-free mass spectrometry-based protein quantification technologies in proteomic analysis. Brief Funct Genomic Proteomic.

[b52] Washburn MP, Wolters D, Yates JR (2001). Large-scale analysis of the yeast proteome by multidimensional protein identification technology. Nat Biotechnol.

[b50] Weinand M, Kramer R, Morbach S (2007). Characterization of compatible solute transporter multiplicity in *Corynebacterium glutamicum*. Appl Microbiol Biotechnol.

[b12] Wolters DA, Washburn MP, Yates JR (2001). An automated multidimensional protein identification technology for shotgun proteomics. Anal Chem.

[b31] Zhang Y, Reckow S, Webhofer C, Boehme M, Gormanns P, Egge-Jacobsen WM, Turck CW (2011). Proteome scale turnover analysis in live animals using stable isotope metabolic labeling. Anal Chem.

